# Eliminating financial disincentives to living kidney donation – a call to action

**DOI:** 10.3389/fmed.2023.1061342

**Published:** 2023-06-30

**Authors:** Karunesh Polireddy, Rebecca L. Crepeau, Abraham J. Matar

**Affiliations:** ^1^Emory University School of Medicine, Atlanta, GA, United States; ^2^Department of Surgery, Emory University School of Medicine, Atlanta, GA, United States

**Keywords:** financial disincentives, overregulation, end stage renal disease, kidney transplantation, living kidney donation

## Abstract

The incidence of end stage renal disease (ESRD) in the United States (US) is increasing each year. The lone curative treatment for ESRD remains kidney transplantation. Despite the demonstrated medical and economic benefits, living donor kidney transplantation (LDKT) only accounts for a small number of kidney transplantations each year. Direct and indirect costs exist that disincentivize potential living kidney donors from coming forward, such as the cost of travel and lodging, risk of death, potential loss of income due to an extended recovery time, and the inability to donate to a relative in the future if needed. Herein, we advocate for policy changes that make living kidney donation (LKD) a financially neutral process thereby incentivizing increased LDKT and mitigating the kidney donor shortage.

## 1. Introduction

The prevalence of end stage renal disease (ESRD) in the United States (US) is rapidly increasing each year. Currently, ESRD effects nearly 750,000 Americans, of which 500,000 receive dialysis, while another 3 million Americans are afflicted with chronic kidney disease. Although life-prolonging, dialysis is not curative and is associated with negative consequences including an increased risk of infectious complications, hospitalizations, and a decreased quality of life (1–5). Kidney transplantation offers the lone curative option for patients with ESRD. Compared to dialysis, kidney transplantation is associated with increased life expectancy, reduced morbidity, and an improved quality of life (6, 7). Living donor kidney transplantation (LDKT) offers several benefits over deceased donor kidney transplantation (DDKT) including superior graft survival rates and a shorter time to transplantation, making LDKT the preferred option when possible (8, 9). Despite this, LDKT still represents only a minority (~25%) of all kidney transplants performed annually (8). Increasing LDKT is crucial, as DDKT alone is not sufficient to meet the growing need for kidneys – this holds true even if 100% of kidneys from medically suitable deceased donors were to be utilized for transplant.

In addition to the clinical benefits afforded by LDKT, economically, it is associated with decreased overall healthcare expenditures when compared to dialysis. Studies estimate that each case of LDKT can save taxpayers between $94,000 and 146,000 compared to the cost to taxpayers for a patient’s dialysis care (10, 11). In the current system of LDKT, the donor’s medical expenses are covered by the recipient’s health insurance. Despite this, there are several financial disincentives that deter potential living kidney donors (LKD) (10). These include direct costs – defined as costs incurred due to the consumption of resources in pre-surgical preparation, completion of the surgery, and patient recovery, as well as indirect costs – defined as incidental costs to the patient as a consequence of surgery and recovery. Direct costs may include transportation, lodging, food, uncovered medical expenses, and other incidental expenses. Indirect costs can include child and dependent-care expenses, lost wages, loss of job stability, use of designated paid time off, and effects on insurability and premium rates (12). McCormick et al. estimated the total direct and indirect cost in the US to LKD resulting from these financial disincentives to be $38,000 per donor (10). A systematic review by Fu et al. of 16 studies based in the US, Canada, United Kingdom, Australia, Norway, and the Netherlands demonstrated that the average cost to LKD ranged from $900 to $19,000 USD, accounting for direct and indirect costs incurred between the pre-donation evaluation and the end of the first post-operative year. Although fewer than half of all LKD sought any financial assistance, 80% of donors reported a financial loss (13). Travel and health services were identified as the most frequently reported financial loss incurred, while loss of income accounted for the largest proportion of financial losses incurred (13).

While the National Organ Transplant Act of 1984 banned the provision of financial incentives for organ donation, the process of living kidney donation should be a financially neutral process, a position asserted by the American Society of Transplantation Living Donor Community of Practice (14). While there have been policies implemented since the early 2000s aimed at providing financial assistance for donors, LKD remains far from a financially neutral process due to the disincentives that exists in the form of both direct and indirect costs to the LKD incurred before, during, and after donation. A call to action is needed at both the individual and societal levels to eliminate financial disincentives to LKD.

## 2. Policy options and implications

### 2.1. Current policies and financial resources

The existing policies and resources available to provide financial relief to LKD are ultimately insufficient, scattered, and underutilized. In 2006, the US Department of Health and Human Services (HHS) announced a commitment to reduce financial barriers to living donation, leading to the inception of the National Living Donor Assistance Center (NLDAC) which is focused on offsetting direct costs incurred through the donation process (15). The NLDAC oversees the living organ donor reimbursement program which receives funding through the Health Resources and Services Administration (HRSA) Reimbursement of Travel and Subsistence Expenses toward Living Organ Donation grants program. In 2019, an executive order expanded reimbursements by the NLDAC beyond travel expenses to include indirect costs such as lost wages and child and elder-care expenses. As of 2022, the NLDAC covers (1) travel expenses, with a controlled value card restricted to pay for transportation, housing, and meals, (2) lost wages for medical evaluation trips (up to 3 days), post-operative recovery (up to 4 weeks), re-hospitalization and post-operative appointments (up to 2 weeks), and (3) dependent care expenses ($504 per week for adult-care expenses and $420 per week for child care expenses). The maximum permitted reimbursement per LKD is $6,000 USD (12).

Eligibility requirements to receive financial reimbursement mandate that the donor be a US citizen or lawfully present resident of the US, maintain a primary residence in the US, and that the donor’s yearly household income should not exceed 350% of the current HHS Poverty Guidelines. As an example, in 2022 a donor would be deemed ineligible to receive financial reimbursement if the donor belonged to a one-person household earning more than $47,565 or a five-person household earning more than $113,645. A donor is also deemed ineligible if they are being provided any reimbursement from the recipient, who may choose to pay for their donors’ travel, lodging, and lost wages per the National Organ Transplant Act. Accordingly, the NLDAC assesses the recipient’s ability to provide financial assistance before approving a donor. A donor is also deemed ineligible if they are provided any reimbursement from the recipient’s insurance company, which may have policies providing travel benefits for their clients’ living donors. Finally, a donor is also ineligible if he or she receives any reimbursement from a state program (12).

Currently, the US NLDAC grant program provides assistance to less than 10% of all LKD each year, and this percentage has increased only marginally since its inception (16, 17). Since its inception, the NLDAC has received ~8,400 applications for funding, of which ~7,500 have been approved. Among the living donors assisted through the NLDAC, the average reimbursement per donor is $2,350 (12). A study by Rodrigue et al. assessed total unreimbursed costs by subtracting total financial assistance received from total costs which equated to roughly $3,200 of non-reimbursed costs on average within the first postoperative year for US LKD (13, 17). Given that the majority of LKD receive no financial assistance, the financial burden to LKD is estimated to be even greater than these numbers indicate.

### 2.2. Short comings of the existing system

Several studies have quantified the mounting costs associated with LKD, acknowledging both direct and indirect costs. Direct costs are defined as any out-of-pocket cash or credit card payments related to hospital expenses, medications, incidental expenses, as well as travel, lodging, and food related to the donation. In one cohort study by Rodrigue et al. (17), 92% of LKD experienced at least one direct cost within the first post-operative year, with 53% reporting three or more direct costs associated with their donation. In the same study, 72% of LKD reported indirect costs such as work absence during recovery, and 29% reported costs associated with an inability to perform necessary services such as dependent care and other activities of daily living (17).

McCormick et al. completed a comprehensive study which estimated costs to LKD in 2017 using historic data from earlier researchers. We have adjusted for the considerable cost-of-living changes over the past half-decade in the United States for the following accepted direct and indirect costs: (1) travel and lodging, (2) loss of income, (3) cost of home and dependent care, (4) risk of dying, (5) pain and discomfort of kidney removal, (6) decrease in long-term quality of life, and (7) concern that a friend or family member may need a kidney in the future (Table 1).

**Table 1:** Direct and indirect costs associated with living-kidney donation.

Costs to LKD	Prior reports (Non-adjusted)	Adjusted for 2022 COL
1. Travel and lodging	$3,122 (McCormick et al)*	$3,744
2. Loss of income	$5,118 (McCormick et al)*	$6,187
3. Cost of home and dependent care	$5,592 (McCormick et al)*	$6,760
4. Risk of death	$1,860 (McCormick et al)* $6,723 (Becker et al) $2,951 (Gaston et al)	$2,248
5. Pain and discomfort	$6,414 (McCormick et al) $5,000 (Gaston et al)*	$7,349
6. Long-term health consequences	$7,910 (McCormick et al)* $10,085 (Becker et al) $23,250 (Gaston et al)	$9,562
7. Future need for donation	$7,728 (McCormick et al)*	$9,342
Total	$37,444	$45,192

*denotes the value used for total and total-adjusted COL calculations.

Based off estimations by McCormick et al. and previous studies and adjusted for cost-of-living changes over the past half-decade, an increase of 20.88% according to the Bureau of Labor Statistics, the estimated direct and indirect costs associated with LKD in 2022 has increased to $43,910 (1, 18).

## 3. Actionable recommendations

### 3.1. Financial disincentives and proposed policy

We advocate for removal of all financial disincentives through policies that can off-set or directly reimburse LKD for direct and indirect costs associated with donation. At the individual level, potential donors should have access to comprehensive information about the costs and benefits of donation, including information about financial support programs that can help offset the donation costs. At the societal level, policies and programs should be developed to support LKD, including reimbursement for lost income and medical expenses, as well as support for long-term follow-up care and monitoring. Additionally, removing regulations that limit financial support for donors, such as restrictions on reimbursement for lost wages, could further reduce the financial barriers to donation.

Held et al. suggested a $45,000-per-kidney compensation for donors, a number similar to our estimated total for the cost of LKD adjusted for 2022 cost of living (19). The study states that the government can expect to spend about $2.6 billion per year in providing $45,000 to all LKD; however, it would in turn save taxpayers $14.1 billion per year from dialysis savings (19). Making living kidney donation a financially neutral process would save taxpayers an estimated $11.5 billion per year, not to mention the societal benefits of improvement in quality of life for ESRD patients and medical benefits of living kidney donations as opposed to cadaveric donations. As of 2022, if a LKD qualifies for financial assistance by meeting its stringent criteria, the NLDAC may cover travel expenses and lodging, lost wages for medical evaluation trips (up to 3 days), post-operative recovery (up to 4 weeks), and re-hospitalization and post-operative appointments (up to 2 weeks), and dependent care expenses (Disincentives 1–3). While the NLDAC partially alleviates LKD of direct costs, it fails to address many of the indirect costs (Disincentives 4–7) (12).

Disincentives 1, 2, and 3 listed in Table 1 include direct costs of $16,691 per LKD accounting for travel and lodging, loss of income, and home- and dependent-care associated with donation and recovery. In 2018, the NLDAC provided financial assistance to only 16% of LKD, in part due to the stringent criteria for those who seek financial assistance. In addition, the maximum reimbursement for those that qualify for financial assistance is $6,000, accounting for roughly one-third of our estimated direct costs (12). We propose the removal of the stringent qualification criteria to include all potential LKD and robust expansion of the NLDAC financial grant program. Finally, removing regulations that limit support to donors, such as restrictions on reimbursement for lost wages, are critically important to further reduce the financial barriers to donation.

Disincentive 4 addresses the small, but important deterrent for many potential LKD — risk of death. The perioperative mortality rate associated with living kidney donation is ~0.03%. Varying based on the statistical value placed on life, McCormick et al., Becker et al., and Gaston et al. have estimated a cost of $1860, $6,723, and $2,951, respectively, per donor to the government (10, 20, 21). We propose a government-sponsored short-term life insurance policy to account for this small, but important indirect cost associated with risk of death that LKD face.

Disincentives 5 and 6 address the short- and long-term financial impact of living kidney donation on potential donors. In the acute post-operative period, complications are not uncommon and include adverse reactions to anesthesia, urinary tract infections, post-operative pneumonia, catheter-associated infections, surgical-site infection, and venous thromboembolic events. Gaston et al. suggested a tax deduction of $10,000 or a nontaxable lump sum payment of $5,000 to offset this indirect cost (20). Based on the study by Gaston et al., we favor a nontaxable lump sum of $7,349 (adjusted for 2022 COL) to LKD rather than a tax deduction because a tax deduction would not provide any benefit to those individuals who do not pay any income tax – which likely represents a significant portion of the population that is likely the most in-need of financial assistance.

The primary long-term cost of LKD is commonly identified as the risk of developing ESRD. Muzaale et al. and Mjøen et al. proposed an increased 15-year risk of developing ESRD at ratios of 7.9 and 11.4, respectively, when comparing donors and non-donors (22, 23). Prior studies have quantified the loss in quality-of life of long-term health consequences as $7,910, $10,085, and $23,250, respectively, per donor to the government (10, 20, 21). To account for the costs associated with both short- and long-term complications associated with LKD, LKD should be eligible for a comprehensive insurance package to include short-term life insurance, disability insurance, and health insurance for long-term medical care.

Disincentive 7 addresses the indirect opportunity cost associated with donating a kidney to an individual on the kidney transplant waiting list and therefore being unable to donate to a relative in the future. Acknowledging the difficulty of quantifying this cost, McCormick et al. estimate the cost at $7,728. A policy ensuring priority placement on the kidney transplant waiting list for any direct relative of a prior LKD could help alleviate this disincentive.

Finally, it is important to note that any reimbursement provided to living donors by a state program must be strictly for direct or indirect costs related to the donation process and cannot be seen as compensation for the organ itself. In addition, donors who receive reimbursement from a state program may need to disclose this information to the transplant center and undergo additional evaluation to ensure that financial considerations do not influence their decision to donate.

## 4. Conclusion

Living donor kidney transplantation remains the gold standard for patients with ESRD given its superior outcomes compared to both DDKT and dialysis. LDKT remains underutilized in part because donation is associated with a net negative financial loss to the donor. Both direct and indirect costs associated with the donation process remain a major barrier for potential donors. Despite the development of programs intended to offset these costs to donors (NLDAC), only a minority of living kidney donors are eligible for existing resources to provide financial assistance due to overregulation.

Moving forward, increasing LDKT will be necessary to meet the growing demand for kidneys. Implementation of policies that strive to make LDKT a financially neutral process, both by providing financial assistance to offset costs as well as removing regulations in place, would increase the number of living kidney donors and should be a top priority for the transplant community.

## Author contributions

KP, RC, and AM conceived and wrote the manuscript. All authors contributed to the article and approved the submitted version.

## Conflict of interest

The authors declare that the research was conducted in the absence of any commercial or financial relationships that could be construed as a potential conflict of interest.

## Publisher’s note

All claims expressed in this article are solely those of the authors and do not necessarily represent those of their affiliated organizations, or those of the publisher, the editors and the reviewers. Any product that may be evaluated in this article, or claim that may be made by its manufacturer, is not guaranteed or endorsed by the publisher.

## Abbreviations

ESRD: End Stage Renal Disease
US: United States
LDKT: Living Donor Kidney Transplantation
DDKT: Deceased Donor Kidney Transplantation
LDK: Living Kidney Donors
USD: US Dollars
HHS: Health and Human Services
NLDAC: National Living Donor Assistance Center
COL: Cost of Living
